# A Directed Continuous Time Random Walk Model with Jump Length Depending on Waiting Time

**DOI:** 10.1155/2014/182508

**Published:** 2014-03-13

**Authors:** Long Shi, Zuguo Yu, Zhi Mao, Aiguo Xiao

**Affiliations:** ^1^Hunan Key Laboratory for Computation and Simulation in Science and Engineering and Key Laboratory of Intelligent Computing and Information Processing of Ministry of Education, Xiangtan University, Xiangtan, Hunan 411105, China; ^2^Institute of Mathematics and Physics, Central South University of Forest and Technology, Changsha, Hunan 410004, China; ^3^School of Mathematical Sciences, Queensland University of Technology, G.P.O. Box 2434, Brisbane, QLD 4001, Australia

## Abstract

In continuum one-dimensional space, a coupled directed continuous time random walk model is proposed, where the random walker jumps toward one direction and the waiting time between jumps affects the subsequent jump. In the proposed model, the Laplace-Laplace transform of the probability density function *P*(*x*, *t*) of finding the walker at position *x* at time *t* is completely determined by the Laplace transform of the probability density function *φ*(*t*) of the waiting time. In terms of the probability density function of the waiting time in the Laplace domain, the limit distribution of the random process and the corresponding evolving equations are derived.

## 1. Introduction

The continuous time random walk (CTRW) theory, which was introduced by Montroll and Weiss [1] to study random walks on a lattice, has been applied successfully in many fields (see, e.g., the reviews [2–4] and references therein).

In continuum one-dimensional space, a CTRW process is generated by a sequence of independent identically distributed (IID) positive waiting times *T*_1_, *T*_2_, *T*_3_,…, and a sequence of IID random jump lengths *X*_1_, *X*_2_, *X*_3_,…. Each waiting time has the same probability density function (PDF) *φ*(*t*), *t* ≥ 0, and each jump length has the same PDF *λ*(*x*) (usually chosen to be symmetric *λ*(*x*) = *λ*(−*x*)). Setting *t*_0_ = 0, *t*_*n*_ = *T*_1_ + *T*_2_ + ⋯+*T*_*n*_ for *n* ∈ *N* and *x*_0_ = 0, *x*_*n*_ = *X*_1_ + *X*_2_ + ⋯+*X*_*n*_, *x*(*t*) = *x*_*n*_ for *t*_*n*_ ≤ *t* < *t*_*n*+1_, we get a microscopic description of the diffusion process [5]. If {*X*_*n*_} and {*T*_*n*_} are independent, the CTRW is called decoupled. Otherwise it is called coupled CTRW [6]. The decoupled CTRW, which is completely determined by mutually independent random jump length and random waiting time, has been widely studied in recent years [3–20].

In some applications it becomes important to consider coupled CTRW [7, 8]. The coupled CTRW can be described by the joint PDF *ϕ*(*x*, *t*) of jump length and waiting time. Because *ϕ*(*x*, *t*)*dx* *dt* is the probability of a jump to be in the interval (*x*, *x* + *dx*) in the time interval (*t*, *t* + *dt*), the waiting time PDF *φ*(*t*) = ∫_−*∞*_^+*∞*^ *ϕ*(*x*, *t*)*dx* and the jump length PDF *λ*(*x*) = ∫_0_^+*∞*^ *ϕ*(*x*, *t*)*dt* can be deduced. Some kinds of couplings and correlations were proposed in [21–25], where the symmetric jump length PDF is chosen. For the coupled CTRW, there exist two coupled forms: *ϕ*(*x*, *t*) = *λ*(*x*)*φ*(*t* | *x*) and *ϕ*(*x*, *t*) = *φ*(*t*)*λ*(*x* | *t*). The first coupled form has been studied sufficiently in many literatures [8, 21–23]. The famous model is Lévy walk. Recently, we considered the second coupled form, discussed the asymptotic behaviors of the coupled jump probability density function in the Fourier-Laplace domain, and derived the corresponding fractional diffusion equations from the given asymptotic behaviors [25].

In this work, we introduce a directed CTRW model with jump length depending on waiting time (i.e., *ϕ*(*x*, *t*) = *φ*(*t*)*λ*(*x* | *t*), *x* > 0, *t* > 0). In our model, the Laplace-Laplace transform [26] of *P*(*x*, *t*) of finding the walker at position *x* at time *t* is completely determined by the Laplace transform of *φ*(*t*). Generally, CTRW processes can be categorised by the mean waiting time *T* = ∫_0_^+*∞*^ *tφ*(*t*)*dt* being finite or infinite. Here we find that the long-time limit distributions of the PDF *P*(*x*, *t*) are a Dirac delta function for finite *T* and a beta-like density for infinite *T*, the corresponding evolving equations are a standard advection equation for finite *T* and a pseudodifferential equation with fractional power of coupled space and time derivative for infinite *T*.

This paper is organized as follows. In Section 2, we introduce the basic concepts of the coupled CTRW. In Section 3, a coupled directed CTRW model is introduced. In Section 4, the limit distributions and the corresponding evolving equations of the coupled directed CTRW model are derived. The conclusions are given in Section 5.

## 2. The Coupled Continuous Time Random Walk

Now we recall briefly the general theory of CTRW [3]. Let *η*(*x*, *t*) be the PDF of just having arrived at position *x* at time *t*. It can be expressed by *η*(*x*′, *t*′) (the PDF of just having arrived at position *x*′ at time *t*′ < *t*) as

(1) η(x,t)=∫−∞+∞dx′∫0+∞dt′η(x′,t′)ϕ(x−x′,t−t′) +δ(x)δ(t).

Then, the PDF *P*(*x*, *t*) with the initial condition *P*(*x*, 0) = *δ*(*x*) can be described by the following integral equation [3]:

(2) $$\begin{array}{c} P\left(x,t\right)=\overset{t}{\underset{0}{\int }}\eta \left(x,t^{\prime }\right)\omega \left(t-t^{\prime }\right)dt^{\prime }, \end{array}$$

where *ω*(*t*) = 1 − ∫_0_^*t*^ *φ*(*τ*)*dτ* is the probability of not having made a jump until time *t*.

Let $\hat{f}(k)$ and $\tilde{g}(s)$ be the transforms of Fourier and Laplace of sufficiently well-behaved (generalized) functions *f*(*x*) and *g*(*t*), respectively, defined by

(3) $$\begin{array}{c} \hat{f}\left(k\right)=ℱ\left\{f\left(x\right);k\right\}=\overset{+\infty }{\underset{-\infty }{\int }}f\left(x\right)e^{ikx}dx,\;k\in R,\\ \tilde{g}\left(s\right)=ℒ\left\{g\left(t\right);s\right\}=\overset{+\infty }{\underset{0}{\int }}g\left(t\right)e^{-st}dt,\;s>s_{0}. \end{array}$$

After using the Fourier-Laplace transforms and the convolution theorems for integral equation (2), one can obtain the following famous algebraic relation [3]:

(4) $$\begin{array}{c} \hat{\tilde{P}}\left(k,s\right)=\frac{1-\tilde{\varphi }\left(s\right)}{s}\cdot \frac{1}{1-\hat{\tilde{\phi }}\left(k,s\right)}. \end{array}$$

## 3. A Coupled Directed CTRW Model

In [23], the author considered a CTRW model with waiting time depending on the preceding jump length, where the author supposed that the PDF of the waiting time is a function of a preceding jump length. In that model, the author introduced a natural “physiological” analogy: after making a jump one needs time to rest and recover. The longer the jump distance is, the longer the recovery and the waiting time needed are. This is an interesting hypothetical physiological example. Motivated by this, we consider a directed CTRW model with jump length depending on the waiting time and give an analogue physiological explanation.

A directed CTRW model with jump length depending on the waiting time can be generated by a sequence of IID positive waiting times *T*_1_, *T*_2_, *T*_3_,…, and a sequence of jumps *X*_1_, *X*_2_, *X*_3_,…; each waiting time has the same PDF *φ*(*t*), *t* ≥ 0. Every time jump has the same direction and each jump length has the same conditional PDF *λ*(*x* | *t*), *x* ≥ 0, which is the PDF of the random walker making a jump of length *x* following a waiting time *t*.

A natural assumption is that the jump length is proportional to the waiting time. So we can take the simplest jump length PDF as *λ*(*x* | *t*) = *δ*(*x* − *vt*), *v* > 0. Without loss of generality, we take *v* = 1 in the following discussion. Setting *t*_0_ = 0, *t*_*n*_ = *T*_1_ + *T*_2_ + ⋯+*T*_*n*_ for *n* ∈ *N* and *x*_0_ = 0, *x*_*n*_ = *X*_1_ + *X*_2_ + ⋯+*X*_*n*_, *x*(*t*) = *x*_*n*_ for *t*_*n*_ ≤ *t* < *t*_*n*+1_, we get a directed CTRW process, where the joint PDF *ϕ*(*x*, *t*) can be expressed by *ϕ*(*x*, *t*) = *φ*(*t*)*δ*(*x* − *t*). A physiological explanation can be made as follows: the walker has a random time for a rest to supplement energy and then makes a jump. The longer the rest time is, the longer the jump length can be.

Since the variable *x* takes positive values in proposed directed CTRW model, it is convenient to replace the Fourier transform for variable *x* in formula (4) with the Laplace transform (i.e.,  $\tilde{f}(k)=ℒ\{f(x);k\}=\int_{0}^{+\infty }f(x)e^{-kx}dx$) to obtain the following Laplace-Laplace relation [26]:

(5) $$\begin{array}{c} \tilde{\tilde{P}}\left(k,s\right)=\frac{1-\tilde{\varphi }\left(s\right)}{s}\cdot \frac{1}{1-\tilde{\tilde{\phi }}\left(k,s\right)}. \end{array}$$

Since

(6) ϕ~~(k,s)=∫0+∞dt∫0+∞ϕ(x,t)e−kx−stdx=∫0+∞dt∫0+∞φ(t)δ(x−t)e−kx−stdx=∫0+∞φ(t)e−(s+k)tdt=φ~(s+k),

(5) is recast into

(7) $$\begin{array}{c} \tilde{\tilde{P}}\left(k,s\right)=\frac{1-\tilde{\varphi }\left(s\right)}{s}\cdot \frac{1}{1-\tilde{\varphi }\left(s+k\right)}. \end{array}$$

The  *n*th (*n* = 1,2) moment of *P*(*x*, *t*) is given by

(8) 〈xn〉(t)=∫0+∞xn(t)P(x,t)dx=(−1)n∂n∂knP~(k,t)|k=0=ℒ−1{1−φ~(s)s·(−1)n∂n∂kn11−φ~(s+k)|k=0}.

In the following section, we will study the possible behaviors of *P*(*x*, *t*) and its *n*th (*n* = 1,2) moment.

## 4. The Limit Distributions of the Coupled Directed CTRW Model

From (7), we can see that the Laplace-Laplace transform of PDF *P*(*x*, *t*) is completely determined by the Laplace transform of the waiting time PDF *φ*(*t*). Usually, the random waiting time is characterized by its mean value *T*. It may be finite or infinite.

For finite mean waiting time *T*, the Laplace transform of *φ*(*t*) is of the form

(9) $$\begin{array}{c} \tilde{\varphi }\left(s\right)=1-sT+o\left(s\right),\;s⟶0. \end{array}$$

Substituting (9) into (7), in the limit (*k*, *s*)→(0,0), we get the asymptotic relation

(10) $$\begin{array}{c} \tilde{\tilde{P}}\left(k,s\right)\sim \frac{1-\left(1-sT\right)}{s}\cdot \frac{1}{1-\left(1-\left(s+k\right)T\right)}=\frac{1}{s+k}. \end{array}$$

After taking the inverse Laplace transforms for (10) about *k* and *s*, we have

(11) $$\begin{array}{c} P\left(x,t\right)=\delta \left(x-t\right). \end{array}$$

For long times

(12) $$\begin{array}{c} \langle x\rangle \left(t\right)=t,\\ \langle x^{2}\rangle \left(t\right)=t^{2}. \end{array}$$

From (10), we get

(13) $$\begin{array}{c} s\tilde{\tilde{P}}\left(k,s\right)-1+k\tilde{\tilde{P}}\left(k,s\right)=0. \end{array}$$

Using $ℒ\{\partial P(x,t)/\partial t;s\}=s\tilde{P}(x,s)-P(x,0)$, $ℒ\{\partial P(x,t)/\partial x;k\}=k\tilde{P}(k,t)-P(0,t)$, initial condition *P*(*x*, 0) = *δ*(*x*), and natural boundary conditions, we obtain the partial differential equation

(14) $$\begin{array}{c} \frac{\partial P\left(x,t\right)}{\partial t}+\frac{\partial P\left(x,t\right)}{\partial x}=0, \end{array}$$

which is the standard advection equation.

In many applications, one needs to consider a long waiting time (i.e., *T* is infinite); it is natural to generalize (9) to the following form:

(15) $$\begin{array}{c} \tilde{\varphi }\left(s\right)=1-s^{\beta }+o\left(s^{\beta }\right),\;s⟶0,\;\;0<\beta \leq 1. \end{array}$$

Inserting (15) into (7), in the limit (*k*, *s*)→(0,0), we get the asymptotic relation

(16) $$\begin{array}{c} \tilde{\tilde{P}}\left(k,s\right)\sim \frac{1-\left(1-s^{\beta }\right)}{s}\cdot \frac{1}{1-\left(1-{\left(s+k\right)}^{\beta }\right)}=\frac{s^{\beta -1}}{{\left(s+k\right)}^{\beta }}. \end{array}$$

After taking the Laplace inverse transform for (16) about *s*, one has

(17) P~(k,t)=t−βΓ(1−β)∗[e−kttβ−1Γ(β)]=∫0te−kττβ−1(t−τ)−βΓ(β)Γ(1−β)dτ,

where we use the formulas *ℒ*{*t* ^*β*−1^; *s*} = Γ(*β*)/*s* ^*β*^ for *β* > 0, $ℒ\{e^{-at}g(t);s\}=\tilde{g}(s+a)$, and $ℒ\{(f*g)(t);s\}=\tilde{f}(s)\tilde{g}(s)$.

According to formula (8) and (17), for long times, one gets

(18) $$\begin{array}{c} \langle x\rangle \left(t\right)=\beta t,\\ \langle x^{2}\rangle \left(t\right)=\frac{\beta \left(\beta +1\right)}{2}t^{2}. \end{array}$$

Then taking the Laplace inverse transform for (17) about *k*, the following form is obtained:

(19) P(x,t)=∫0tδ(x−τ)τβ−1(t−τ)−βΓ(β)Γ(1−β)dτ=xβ−1(t−x)−βΓ(β)Γ(1−β),

which is the density of a random variable *tB*, where *B* has a Beta distribution with parameters *β* and 1 − *β*.

From (17), we can also obtain

(20) $$\begin{array}{c} {\left(s+k\right)}^{\beta }\tilde{\tilde{P}}\left(k,s\right)=s^{\beta -1}, \end{array}$$

which leads to the pseudodifferential equation [27, 28]

(21) $$\begin{array}{c} {\left(\frac{\partial }{\partial t}+\frac{\partial }{\partial x}\right)}^{\beta }P\left(x,t\right)=\delta \left(x\right)\frac{t^{-\beta }}{\Gamma \left(1-\beta \right)} \end{array}$$

with a coupled space-time fractional derivative operator on the left-hand side.

Equation (21) is useful to model flow in porous media and other physical systems characterized by a link between the waiting time and the jump length.

## 5. Conclusions

In this work, we introduce a directed CTRW model with jump lengths depending on waiting times. By the Laplace-Laplace transform technique, we find that the PDF *P*(*x*, *t*) is determined only by the waiting times PDF *φ*(*t*). For finite and infinite mean waiting time, we deduce the limit distributions of *P*(*x*, *t*) from the asymptotic behaviors of *φ*(*t*) in the Laplace domain, respectively. The corresponding evolving equations are also derived. For finite mean waiting time, the limit behavior of the PDF *P*(*x*, *t*) is governed by a standard advection equation. For infinite mean waiting time, the limit behavior of the PDF *P*(*x*, *t*) is governed by a pseudodifferential equation with coupled space-time fractional derivative. We also calculate the first-order moment 〈*x*〉(*t*) and the second-order moment 〈*x* ^2^〉(*t*) of *P*(*x*, *t*). An interesting phenomenon is obtained: there exist the relations 〈*x*〉(*t*) ~ *t*, 〈*x* ^2^〉(*t*) ~ *t* ^2^, whether the mean waiting time is finite or not.
